
JOURNAL INFORMATION
==============================
NLM Title Abbreviation: ScientificWorldJournal
Journal ID: ScientificWorldJournal
NLM Journal ID: 101131163
Journal ID: TSWJ

The Scientific World Journal

ISSN: 2356-6140
EISSN: 1537-744X
Publisher: Wiley

ARTICLE INFORMATION
==============================
PMCID: PMC6084019
PMID: 30147546
DOI: 10.1100/tsw.2001.111
Article ID: 196237
Article version: 1
Subjects: Short Report

Aging Enhances the Activation of the Permeability Transition Pore in Mitochondria

Rottenberg Hagai *
Mather Michael
Department of Microbiology and Immunology, MCP Hahnemann University School of Medicine, 245 N. 15th Street, M.S. 435, Philadelphia, PA 19102, USA
*Hagai Rottenberg: hagai.rottenberg@drexel.edu
Publication date: 2001
Electronic publication date: 2001 Jun 5
Volume: 1
First page: 71
Last page: 71
Copyright: Copyright © 2001 Hagai Rottenberg and Michael Mather.
Copyright year: 2001
License: This is an open access article distributed under the Creative Commons Attribution License, which permits unrestricted use, distribution, and reproduction in any medium, provided the original work is properly cited.
License URL: https://creativecommons.org/licenses/by/3.0/

Funding: National Institutes of Health AG13799

==============================
Miami *Nature Biotechnology* Short Reports
*TheScientificWorld* (2001) 1(S3), 71SR
ISSN 1532-2246; DOI 10.1100/TSW.2001.111

### AGING ENHANCES THE ACTIVATION OF THE PERMEABILITY TRANSITION PORE IN MITOCHONDRIA

Hagai Rottenberg* and Michael Mather

Department of Microbiology and Immunology, MCP Hahnemann University School of Medicine, 245 N. 15th Street, M.S. 435, Philadelphia, PA 19102
* hagai.rottenberg@drexel.edu

**INTRODUCTION.** Mitochondrial dysfunction is believed to be a major cause of cell death and tissue degeneration in aging. However, it is not known which of the many mitochondrial defects that are associated with aging is critical for cell survival. The mitochondrial Permeability Transition Pore (PTP) is known to play a critical role in apoptosis and necrosis (1). We have found that aging enhances the activation of PTP in mouse lymphocytes, and in isolated mitochondria from liver and brain, suggesting that this activation causes cell death and tissue degeneration in aging (2,3). We also show that the attenuation of calcium signaling in T cells from old mice results from the enhancement of PTP activation.

**METHODS.** Mitochondria were isolated from liver and brain of young (3-4 mo.) and old (20 mo.) mice, and PTP activation was assayed with the calcium indicator arsenazo III. T cells were isolated from the spleen of young and old mice and calcium signaling was assayed by flow cytometry with the calcium indicator fluo-3. PTP activation in T cells was assayed by flow cytometry with the mitochondrial membrane potential ( $_m$) indicator $DiOC_6(3)$.

**RESULTS.** Calcium overloading induces PTP activation in isolated mitochondria from liver and brain, which release the accumulated calcium. The activation of PTP is inhibited by cyclosporin, A which allow the mitochondria to accumulate large amounts of calcium. We determined the threshold for PTP activation in liver and brain mitochondria from young and old mice. About 40% more calcium was required to trigger PTP in liver mitochondria from young mice compared to old mice ($P<0.00002$, n=5). Moreover, cyclosporin A was more effective in protecting mitochondria from calcium overloading in old mice ($P<0.001$, n=5). These results indicate that there is enhanced activation of PTP in liver mitochondrial from old mice. Similar results were obtained with brain mitochondria. The threshold for calcium activation of PTP is higher in brain mitochondria than in liver mitochondria and the difference between young and old mice is smaller, but still highly significant ($P<0.006$, n=5).

Calcium signaling, including ionomycin-induced signaling, is greatly attenuated In T lymphocytes from old mice (4). PTP is highly activated in isolated T cells from old mice resulting in very low $_m$. Cyclosporin A as well as the cyclosporin derivative MV-Cs (which does not inhibit calcineurin), restored $_m$ in T cells from old mice, and also restored the ionomycin-induced sustained elevation of cell free calcium. Since it is now established that $_m$ is critical for calcium signaling in T cells (5), these results suggest that the aging-induced attenuation of calcium signaling in T cells is the direct result of the enhanced activation of PTP. We also show that the mitochondria in T cells from old mice generate more superoxide and are in a more oxidized state, which may be the direct cause of the enhanced activation of PTP.

**DISCUSSION.** Aging induces a large array of mitochondrial defects: mtDNA deletions and mutations, oxidative damage to many enzymes of intermediate metabolism and oxidative phosphorylation, and oxidative damage to membrane lipids. However, it is not clear which of these defects, if any, is critical for cell damage in aging. PTP activation is known to induce cell death and therefore our finding of aging-induced enhanced activation of PTP suggest that this is the direct cause of cell and tissue damage in aging. Moreover, the finding that an enhanced activation of PTP causes attenuation of calcium signaling in T cells from old mice suggest that other more subtle defects in aging are also caused by the enhanced activation of PTP.

**ACKNOWLEDGEMENT.** Supported by NIH grant AG13799.

**REFERENCES.**

1. Kroemer, G., Dallaporta, B., and Rescfe-Rigon, M. (1998) Annu. Rev. Physiol. 60, 619-642
2. Rottenberg, H. and Wu, S. (1997) Biochem. Biophys. Res. Commun. 240, 68-74
3. Mather, M. and Rottenberg, H. (2000) Biochem. Biophys. Res. Commun. 273, 603-608
4. Miller, R.A. (1996) Life Sci. 59, 469-475
5. Hoth, M., Button, D.C., and Lewis, R.S. (2000) Proc. Natl. Acad. Sci. U S A 97, 10607-10612
